# Relatively Lower FT3 Levels Are Associated with Impaired Quality of Life in Levothyroxine-Treated Patients with Hashimoto Thyroiditis

**DOI:** 10.1155/2022/1918674

**Published:** 2022-03-09

**Authors:** Zhijun Cui, Xiaoyu Ding, Nannan Bian, Xiaona Chang, Jiaxuan Wang, Yu An, Jia Liu, Guang Wang

**Affiliations:** Department of Endocrinology, Beijing Chao-Yang Hospital, Capital Medical University, Beijing 100020, China

## Abstract

**Objective:**

Patients with Hashimoto thyroiditis (HT) frequently have some complaints despite achieving euthyroidism after levothyroxine (LT4) treatment. This study aimed to investigate the relevant factors affecting the quality of life (QoL) in euthyroid HT patients after LT4 treatment.

**Methods:**

In this case-control study, 133 participants with HT were included. They were divided into two groups: 64 euthyroid HT subjects (control group) and 69 HT patients were rendered euthyroid by LT4 treatment (well-controlled group). QoL was measured with the Thyroid-Related Patient-Reported Outcome (ThyPRO-39) questionnaire.

**Results:**

Both study groups were well matched with respect to gender, age, BMI, euthyroidism, and thyroid antibodies (TPOAb and TGAb). Compared with the control group, the well-controlled group had lower FT3 (*P* < 0.01) levels. Of note, QoL was impaired on all scales in the well-controlled group. Moreover, ThyPRO-39 scores among the well-controlled group were significantly higher (worse) than the control group in all scales. Regarding the composite scale, its score was related to FT3 (*r* = −0.176, *P*=0.043) but not to FT4 and TSH levels. Further logistic regression analysis revealed FT3 was significantly associated with elevated composite QoL [0.128 (0.029–0.577), *P* < 0.01] after adjustment of potential confounders.

**Conclusion:**

Relatively lower FT3 concentrations, even within the normal reference range, were related to impaired QoL in HT patients treated with LT4. This finding supports the great value of FT3 in clinical decision-making on dose adequacy.

## 1. Introduction

Hashimoto thyroiditis (HT), also called chronic lymphocytic thyroiditis, is a common autoimmune disease characterized by an enlarged thyroid gland, lymphocytic infiltration of the thyroid parenchyma, and increased circulating antibodies to thyroid antigens [1]. HT is now considered the most common cause of primary hypothyroidism [2, 3], and its incidence has increased rapidly in recent years [4]. The treatment of choice for HT is levothyroxine (LT4) replacement therapy, adjusting the dosage to normalize thyroid function as needed [5]. In recent years, the paradigm for the treatment of hypothyroidism has shifted from restoring physiological imbalances towards prolonging life and improving quality of life (QoL), which can be assessed objectively using a standardized and validated Thyroid-Specific Patient-Reported outcome questionnaire (ThyPRO-39) [6–8]. Of note, in daily clinical practice, we often notice that patients with hypothyroidism have discomfort and complaints, even though they have biochemical euthyroidism after LT4 treatment.

Until now, these residual symptoms remain puzzling, although several possible explanations have been proposed, including the presence of thyroid autoimmunity, the coexistence of other autoimmune diseases, the awareness of having a chronic disease, and the phenomenon of weight gain [9–12]. Evidence is mounting that treatment of hypothyroidism with LT4 does not normalize triiodothyronine (T3) levels in all tissues [13–15], nor does it reproduce the serum thyroid hormone profiles of native euthyroidism [16, 17]. Therefore, the aim of this study was to assess self-reported QoL as a measure of treatment satisfaction and explore its potentially related factors among HT patients with well-controlled hypothyroidism, using the thyroid-specific QoL questionnaire ThyPRO-39.

## 2. Materials and Methods

### 2.1. Participants and Study Design

From June 2020 to August 2021, 133 patients (18–75 years) with HT attending the endocrinological outpatient clinics at Beijing Chao-yang Hospital Affiliated to Capital Medical University were recruited. All participants were divided into two groups: 64 euthyroid HT subjects (control group) and 69 HT patients were rendered euthyroid by LT4 treatment (well-controlled group). Before inclusion in the study, they all were stable in biochemical balances for at least 3 months, and the well-controlled HT patients took the stable dose of LT4 for no less than 3 months. Exclusion criteria were diabetes, chronic heart, pulmonary, liver or kidney diseases, pregnancy or lactation, disorders in thalamus and pituitary, history of thyroid surgery, cancer (including thyroid carcinoma), psychiatric illness (depression or anxiety disorders), dementia, anemia, systemic autoimmune diseases, myopathy, chronic diarrhea, electrolyte disturbance, or any condition that would result in compromised QoL and use of medications that influence thyroid function.

HT was diagnosed as an increase in at least one circulating thyroid antibody, mainly anti-thyroperoxidase antibodies (TPOAb) and anti-thyroglobulin antibodies (TGAb), along with sonographic signs of chronic inflammation (a decreased echogenicity of the thyroid parenchyma) on a thyroid ultrasound [1]. This study was conducted following the ethical principles of Declaration of Helsinki. The protocol has been approved by the Ethics Committee of our hospital. Informed consent was obtained from all enrolled participants.

### 2.2. Measurements

Demographic and clinic information including age, sex, anthropometric measurements, duration of hypothyroidism, history of thyroid disorders, drugs, and drug dose were collected using a standard questionnaire. BMI was calculated as weight divided by height squared (kg/m^2^). Blood samples were collected after overnight fasting in the morning. Free triiodothyronine (FT3), free thyroxine (FT4), total triiodothyronine (TT3), total thyroxine (TT4), thyroid-stimulating hormone (TSH), and thyroid antibodies (TPOAb and TGAb) were measured by electrochemiluminescence immunoassay (ECLIA) using an Abbott Architect i2000 (Abbott Diagnostics, Abbott Park, IL, USA). Reference ranges are 2.3–4.2 pg/ml for FT3, 0.89–1.76 ng/dl for FT4, 0.6–1.81 ng/ml for TT3, 4.5–10.9 *μ*g/dl for TT4, 0.55–4.78 *μ*IU/ml for TSH, and <60 U/ml for thyroid antibodies.

### 2.3. Patient-Reported Outcome ThyPRO

Health-related QoL was examined with the short version of ThyPRO (ThyPRO-39), a comprehensive and thoroughly validated questionnaire [7]. The instrument consists of 39 items, summarized into 12 scales and 1 single-item scale, examining overall QoL. It covers three major areas: physical symptoms (including goiter, hyperthyroid, hypothyroid, and eye symptoms), mental health (including tiredness, cognition, anxiety, depression, and emotional susceptibility), and aspects impacted by thyroid diseases (including impaired social life, daily life, cosmetic concern, and overall quality of life). Each item is a response to a 0–4 Likert scale from 0 = “no symptoms/problems” to 4 = “severe symptoms/problems,” referring to the past 4 weeks. The total score of each scale ranges from 0 to 100, with higher scores indicating worse health status. Additionally, a composite score, based on items apart from the physical symptom score, was also computed for assessing mental and social well-being and function.

### 2.4. Statistical Analysis

The Kolmogorov–Smirnov test was performed to examine the distribution of continuous variables. Data were expressed as the mean ± standard deviation or median (interquartile range) for continuous data and as frequencies for categorical data. Comparisons between the two groups were analyzed by an independent *t*-test for normally distributed variables or a Mann–Whitney *U* test for skewed-distribution variables. The chi-square test was used for categorical variables. Pearson or Spearman correlation was used to assess correlation. Logistic regression analysis was performed to find the determinations of QoL in HT patients. All analyses were conducted using IBM SPSS 26.0 (IBM Corp., Armonk, New York, USA). A two-tailed *P* < 0.05 was considered statistically significant.

## 3. Results

### 3.1. Patients' Characteristics

The clinical characteristics of all participants are shown in Table 1. Both study groups were well matched with respect to gender, age, BMI, euthyroidism, and thyroid antibodies (TPOAb and TGAb). Most subjects in both groups were female, and there were no significant differences in TT4 and TSH levels between two groups. As expected, well-controlled group had higher FT4 levels (*P* < 0.05), lower FT3 levels (*P* < 0.01), and TT3 levels (*P* < 0.01), compared with the control group.

### 3.2. Patients' QoL

**Figure 1:**
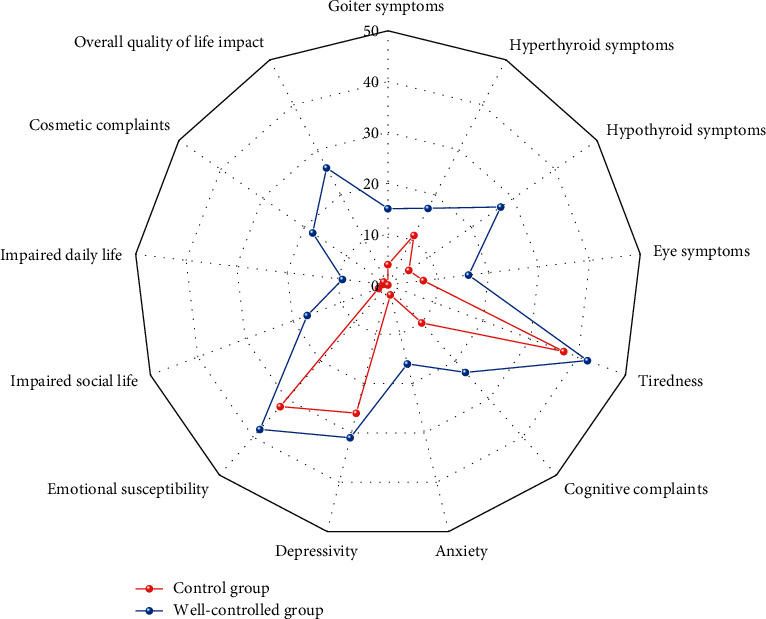
Radar plot of mean ThyPRO scores evaluated in both groups. Each scale ranges from 0–100, with higher scores indicating poorer quality of life.

The results of the ThyPRO-39 questionnaire are summarized in Table 1 and Figure 1. QoL was impaired on all scales in the well-controlled group. In both groups, the most influenced domains of the questionnaire were tiredness, emotional susceptibility, and depression. Moreover, ThyPRO-39 scores among the well-controlled group were significantly higher (worse) than controls in all scales (*P* < 0.05).

### 3.3. QoL and Thyroid Hormones

**Figure 2:**
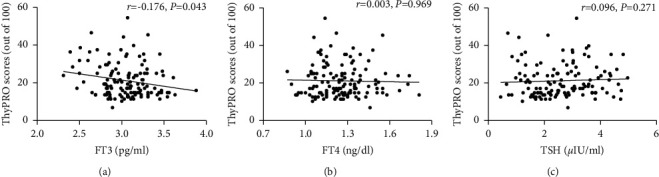
The correlation of FT3 (a), FT4 (b), and TSH (c) levels and ThyPRO composite scores in all patients with HT.

Table 2 shows the correlations between the scores of each scale and the FT3, FT4, or TSH values in the whole study population. The levels of FT3 were negatively associated with hyperthyroid symptoms (*r* = −0.204, *P*=0.018), hypothyroid symptoms (*r* = −0.182, *P*=0.036), eye symptoms (*r* = −0.174, *P*=0.045), cognitive complaints (*r* = −0.251, *P*=0.004), anxiety (*r* = −0.187, *P*=0.031), and cosmetic complaints scales (*r* = −0.337, *P* < 0.001). Of note, the TSH levels were only slightly positively correlated with the tiredness scale (*r* = 0.177, *P*=0.042). Besides, there was no significant correlation between the scores of each scale and serum FT4 levels. Regarding the composite scale, its score was related to FT3 (*r* = −0.176, *P*=0.043) but not to FT4 and TSH levels (Figure 2).

### 3.4. Main Factors Affecting QoL

To determine the effect of thyroid-related variables on QoL, logistic regression analysis was performed across the whole cohort. As shown in Table 3, FT3 levels were significantly associated with the elevated composite QoL (0.193 (0.052–0.712), *P* < 0.05)) without adjustment of potential confounders. In model 2, with adjustment for sex, age, and BMI, FT3 levels remained significantly related to elevated composite QoL (0.157 (0.040–0.613), *P* < 0.01). In model 3, with further adjustment to TPOAb, TGAb, FT4, and TSH, we found FT3 levels remained independently associated with elevated composite QoL (0.128 (0.029–0.577), *P* < 0.01). However, no significant association was observed between composite QoL with FT4 and TSH.

## 4. Discussion

Patients treated with LT4 frequently have discomfort and complaints, although their TSH value is within the normal laboratory range. However, the reason behind this condition is unclear. In the present study, the well-controlled group had lower FT3 concentrations and worse QoL as compared with the control group, using the disease-specific instrument ThyPRO-39. In particular, a positive association was observed between FT3 levels, but not FT4 and TSH, and a composite scale for assessing mental and social well-being. These results provide an insight into the clinical significance of FT3 on treatment satisfaction in HT patients.

Studies have shown that hypothyroidism is relevant to impaired physical, mental, and general aspects of QoL [18, 19]. LT4 monotherapy replacement therapy is the treatment of choice. The treatment target is to normalize TSH levels and relieve symptoms [3]. Nevertheless, many biochemically well-controlled patients with LT4-treated hypothyroidism have persistent complaints. Results from two cross-sectional studies showed that, compared with general population samples, there were small but significant impairments in QoL among patients treated with LT4 [20, 21]. Quran et al. found that all domains (using ThyPRO) were impacted in patients receiving LT4 therapy [22]. Moreover, a prospective cohort study observed that full recovery was not obtained in patients with autoimmune hypothyroidism, despite most aspects improving after 6 months of LT4 therapy [23]. Overall, consistent with findings reported in our study, previous research suggested that QoL was greatly improved, but not always normalized, by LT4 treatment in patients with hypothyroidism.

Several possible explanations for those persistent complaints have been proposed. Results from a prospective cohort study showed that euthyroid female patients with HT had a high symptom load, which was unrelated to hypothyroidism [24]. Another cross-sectional study also found that autoimmunity per se was a determinant to lead to residual symptoms, using a state-of-the-art QoL questionnaire ThyPRO [9]. However, Morón-Díaz et al. observed that higher TSH levels within the normal range were associated with worse QoL in subjects with well-controlled hypothyroidism [25]. Unlike ours, their study included subjects with primary hypothyroidism of any cause, psychiatric illness (depression or anxiety disorders), and the current use antianxiety and/or antidepressant drugs, which might affect the QoL. Additionally, a double-blind randomized cross-over trial showed that a decreased QoL in LT4-treated patients with hypothyroidism was associated with a higher BMI [12]. These inconsistent or contradictory results might be due to significant differences in study designs, patient population, and control population. Thus, we chose the control group as euthyroid HT patients without receiving LT4 treatment rather than healthy subjects, and both groups were strictly matched for sex, age, BMI, and thyroid antibodies. This can rule out the effect of thyroid autoimmunity and other potential confounding factors, including chronic disease self-awareness and the placebo effect of health care, even without LT4 therapy.

Since 2009, the advent of a comprehensive and thoroughly validated thyroid-specific assessment tool for patients with thyroid diseases, ThyPRO, has called for studies measuring the associations between clinical variables and QoL [6–8]. In the present study, we used the short version of ThyPRO-39, which has good applicability in daily clinical practice [7]. In our study, we observed that none of the ThyPRO-39 domains had a significant relationship with FT4 levels, TSH levels were only slightly positively correlated with the tiredness scale, while lower FT3 concentrations were more strongly associated with greater deterioration of QoL. Importantly, the composite scale as a comprehensive measure of thyroid-specific QoL was negatively correlated to the level of FT3, rather than FT4 and TSH. Of note, further logistic analysis clearly showed that lower FT3 levels were more strongly related to greater deterioration of QoL in all patients after adjusting for potential confounders. In contrast to results of our study, Saravanan et al. found that QoL was associated with FT4 concentrations among patients treated with LT4 using the General Health Questionnaire and Thyroid Symptoms Questionnaire (TSQ), but no relationship with FT3 was detected [26]. These findings are not directly comparable with ours due to the different ways of assessing QoL. TSQ has not been validated, which may explain why they did not find the effect of FT3 on patients' QoL. Moreover, Michaelsson et al. found that higher FT3 values were associated with worse QoL, which could be caused by a type 1 error [27]. Finally, this study also observed that the well-controlled group had a high score in the hyperthyroid symptom scale and that the levels of FT3 were negatively associated with hyperthyroid symptoms. This may be because hyperthyroid symptoms mimic those of anxiety. Previous studies showed worse anxiety scores on hospital depression and anxiety scales after treatment with LT4 [12, 28], which was similar to our study. Patients treated with LT4 had relatively lower FT3 and treated patients were inherently more anxious and conscious about their illness leading to a greater psychological burden, which may explain why FT3 levels were negatively associated with hyperthyroid symptoms in this study.

As discussed earlier, LT4 doses sufficient to maintain TSH within the normal range could not ensure adequate circulating FT3 levels in most patients [16, 17, 29–31]. This might be the reason for the lack of symptom relief in well-controlled patients with hypothyroidism. However, LT4 treatment could restore FT3 levels to native euthyroid has always been a controversial point [32]. FT3, the active form of thyroid hormones, is partly (about 20%) derived from direct thyroidal secretion. However, in LT4-treated patients with hypothyroidism, all circulating T3 is converted by deiodination via deiodinase 1 (D1) and deiodinase 2 (D2) pathways. The role played by D2 is predominant, mainly because of its high affinity for T4 [33]. Animal studies suggested that D2 was negatively regulated by T4 and inactivated by WSB-1, which is ubiquitination [34]. Interestingly, D2 activity is lost much more slowly in the hypothalamus, leading to TSH levels being normalized before circulating T3 concentrations are fully restored. This may pathophysiologically explain our findings on the association between FT3 levels and residual symptoms.

Hence, this demands a greater focus on symptomatic patients and reconsideration of treatment modalities, such as the choice of LT4/liothyronine (LT3) combination therapy [35–38]. Nevertheless, many symptoms are nonspecific in older people, QoL scores may not correlate clearly with thyroid function status, and there may be no indication for combined therapy [39].

This study has several limitations. First, our research was a cross-sectional design with a relatively small sample size. Second, the baseline QoL of patients treated with LT4 was not available, which may have allowed a longitudinal comparison of QoL before and after LT4 treatment. Third, there were no standard ThyPRO data on the general population. Fourth, the clinical score, the systolic and diastolic performance, and the neurological and neuropsychological evaluations were not measured, which might offer a better correlation with QoL.

In conclusion, compared with controls, worse QoL was observed among euthyroid HT patients after LT4 treatment, which was independently related to lower FT3 levels, even within the normal range. Our study provides clinical significance for this disequilibrium that LT4 treatment cannot ensure adequate FT3 concentrations despite the biochemical normalization of TSH. Thus, LT4/LT3 combination therapy might be more beneficial for those hypothyroid patients who have persistent complaints.

## Figures and Tables

**Table 1:** Comparison of clinical characteristics and ThyPRO scores between the two groups.

Variable	Control group, *n* = 64	Well-controlled group, *n* = 69	*P*
Age, years	45.94 ± 12.76	45.10 ± 12.24	0.700
Female/male, n	60/4	61/8	0.282
BMI, kg/m^2^	23.87 ± 3.86	23.81 ± 3.91	0.929
TT3, ng/ml	1.16 (1.01, 1.32)	1.03 (0.94, 1.26)	**0.006**
TT4, *μ*g/dl	8.90 (7.63, 10.08)	8.55 (7.70, 9.68)	0.537
FT3, pg/ml	3.13 ± 0.27	2.97 ± 0.27	**0.001**
FT4, ng/dl	1.21 ± 0.16	1.28 ± 0.19	**0.016**
TSH, *μ*IU/ml	2.63 ± 0.98	2.55 ± 1.13	0.692
TPOAb, U/ml	785.65 (164.93, 1855.28)	1156.7 (118.3, 3174.5)	0.447
TGAb, U/ml	223.2 (84.23, 400.2)	148.0 (79.63, 481.58)	0.860
ThyPRO			
Goiter symptoms	2 (2, 2)	10 (10, 15)	**<0.001**
Hyperthyroid symptoms	8 (2, 18)	18 (8, 23)	**<0.001**
Hypothyroid symptoms	6.25 (0, 6.25)	25 (12,5, 37.5)	**<0.001**
Eye symptoms	8 (1, 8)	14 (8, 22.5)	**<0.001**
Tiredness	33 (25, 42)	42 (33, 50)	**0.021**
Cognitive complaints	1 (1, 14)	21 (7, 37)	**<0.001**
Anxiety	1 (1, 1)	10 (1, 26)	**<0.001**
Depression	29 (22, 29)	29 (22, 37)	**0.018**
Emotional susceptibility	28 (28, 36)	36 (28, 52)	**0.009**
Impaired social life	0 (0, 0)	17 (0, 25)	**<0.001**
Impaired daily life	0 (0, 0)	0 (0, 15)	**<0.001**
Cosmetic complaints	1 (1, 1)	1 (1, 28)	**<0.001**
Overall quality of life impact	0 (0, 0)	25 (0, 25)	**<0.001**
Composite scale	14.77 (13.64, 17.05)	23.86 (18.75, 31.82)	**<0.001**

Data are means ± s.d. unless indicated otherwise. Bold indicates *P* value <0.05.

**Table 2:** Correlations of FT3, FT4, TSH, and ThyPRO scores in all patients with HT.

	FT3		FT4		TSH	
	*r*	*P*	*r*	*P*	*r*	*P*
Goiter symptoms	−0.144	0.099	0.142	0.104	0.085	0.330
Hyperthyroid symptoms	−0.204^*∗*^	0.018	0.024	0.788	0.090	0.304
Hypothyroid symptoms	−0.182^*∗*^	0.036	0.079	0.368	−0.062	0.478
Eye symptoms	−0.174^*∗*^	0.045	−0.097	0.266	−0.017	0.850
Tiredness	−0.152	0.082	−0.039	0.654	0.177^*∗*^	0.042
Cognitive complaints	−0.251^∗∗^	0.004	−0.051	0.559	0.055	0.531
Anxiety	−0.187^*∗*^	0.031	0.082	0.350	−0.001	0.993
Depression	0.022	0.804	−0.055	0.531	0.097	0.268
Emotional susceptibility	0.027	0.755	0.003	0.970	0.000	0.997
Impaired social life	−0.066	0.450	0.095	0.275	−0.023	0.797
Impaired daily life	−0.163	0.061	−0.012	0.891	−0.048	0.584
Cosmetic complaints	−0.337^∗∗^	<0.001	−0.046	0.595	0.041	0.638
Overall quality of life impact	−0.148	0.088	0.042	0.635	0.064	0.464
Composite scale	−0.176^*∗*^	0.043	0.003	0.969	0.096	0.271

^*∗*^ *P* < 0.05;^∗∗^*P* < 0.01

**Table 3:** Logistic regression analysis for the association of FT3, FT4, TSH, and QoL in all patients with HT.

	Model 1		Model 2		Model 3	
	OR (95% CI)	*P*	OR (95% CI)	*P*	OR (95% CI)	*P*
FT3	0.193 (0.052–0.712)	0.014	0.157 (0.040–0.613)	0.008	0.128 (0.029–0.577)	0.007
FT4	1.217 (0.181–8.196)	0.840	0.993 (0.141–7.014)	0.994	1.781 (0.217–14.603)	0.591
TSH	1.058 (0.803–1.393)	0.689	1.079 (0.815–1.428)	0.595	1.407 (0.971–2.083)	0.071

Model 1: unadjusted; Model 2: adjusted for age, sex, and BMI; Model 3: Model 2 + TPOAb and TGAb, and FT3, FT4, and TSH adjusted for each other.

## Data Availability

The data generated during the current study are available from the corresponding author upon reasonable request.

## Supplementary Materials

Supplementary Materials Supplementary Table 1: comparison of clinical characteristics and ThyPRO scores between the two subgroups in the well-controlled group.
