# Supplementary material for: Relatively Lower FT3 Levels Are Associated with Impaired Quality of Life in Levothyroxine-Treated Patients with Hashimoto Thyroiditis
Source: Int J Endocrinol. 2022 Mar 9;2022:1918674. doi: 10.1155/2022/1918674 (PMC8926544; doi:10.1155/2022/1918674)
Supplement: Supplementary Materials — Supplementary Table 1: comparison of clinical characteristics and ThyPRO scores between the two subgroups in the well-controlled group. [file 1918674.f1.docx]

**Supplementary Table 1.** Comparison of clinical characteristics and ThyPRO scores between the two subgroups in well-controlled group.

| **Variable** | **Younger n = 36** | **Older**  **n = 33** | ***P*** |
| --- | --- | --- | --- |
| Age, years | 35.19 ± 5.83 | 55.91 ± 7.10 | **< 0.001** |
| Female/male, n | 33/3 | 28/5 | 0.373 |
| BMI, kg/m^2^ | 23.83 ± 4.48 | 23.78 ± 2.65 | 0.957 |
| TT3, ng/ml | 1.00 (0.90, 1.15) | 1.08 (0.95, 1.32) | 0.106 |
| TT4, μg/dl | 8.50 (7.08, 9.43) | 8.65 (7.73, 10.18) | 0.428 |
| FT3, pg/ml | 3.00 ± 0.26 | 2.94 ± 0.28 | 0.360 |
| FT4, ng/dl | 1.30 ± 0.16 | 1.27 ± 0.12 | 0.458 |
| TSH, μIU/ml | 2.58 ± 1.12 | 2.52 ± 1.16 | 0.842 |
| **ThyPRO** | | | |
| Goiter symptoms | 10 (10, 20) | 10 (10, 15) | 0.750 |
| Hyperthyroid symptoms | 13 (8, 18) | 18 (8, 28) | 0.169 |
| Hypothyroid symptoms | 25 (7.81, 37.5) | 18.8 (12,5, 40.6) | 0.681 |
| Eye symptoms | 8 (8, 14) | 20 (8, 28.5) | **0.015** |
| Tiredness | 42 (33, 50) | 50 (33, 58) | 0.063 |
| Cognitive complaints | 14 (7, 29) | 21 (14, 40.5) | 0.051 |
| Anxiety | 14 (1, 26) | 10 (1, 30) | 0.666 |
| Depressivity | 29 (22, 37) | 29 (22, 37) | 0.626 |
| Emotional susceptibility | 44 (30, 52) | 28 (24.5, 40) | **0.011** |
| Impaired social life | 17 (0, 25) | 17 (0, 21) | 0.276 |
| Impaired daily life | 15 (0, 15) | 0 (0, 15) | 0.340 |
| Cosmetic complaints | 21 (1, 28) | 1 (1, 28) | 0.214 |
| Overall quality of life impact | 25 (6.25, 50) | 25 (0, 25) | 0.358 |
| Composite scale | 25 (20.45, 29.55) | 23.86 (17.05, 34.66) | 0.718 |

Data are means ± s.d. unless indicated otherwise. Bold indicates *P* value < 0.05.
